# A case report of delayed persistent third-degree atrioventricular block 7 years after eccentric umbrella occlusion of a perimembranous ventricular septal defect in infancy

**DOI:** 10.3389/fped.2025.1591928

**Published:** 2025-06-04

**Authors:** Lihui Wei, Decai Zeng, Liuliu Huang, Xiangjie Luo, Ji Wu

**Affiliations:** ^1^ Department of Ultrasonic Medicine, The First Affiliated Hospital of Guangxi Medical University, Nanning, China; ^2^ Department of Cardiothoracic Surgery, The First Affiliated Hospital of Guangxi Medical University, Nanning, China

**Keywords:** perimembranous ventricular septal defect, trans-thoracic occlusion, eccentric occluder, delayed atrioventricular block, device-related complications

## Abstract

This article reports an 8-year-old female patient who underwent trans-thoracic small-incision interventional Pm-VSD occlusion with an eccentric umbrella (defect diameter 10 mm, occluder waist diameter 12 mm) at 4 months of age (July 2017). Seven years after the operation (October 2024), she gradually developed third-degree atrioventricular block. Despite the removal of the occluder and ventricular septal defect repair combined with steroid pulse therapy, the conduction abnormality persisted. This case, with a 7 - year disease course evolution, provides in - depth insights into the development process of delayed complete atrioventricular block (CAVB) following eccentric umbrella occlusion in infancy and early childhood, particularly for high - risk patients with occlusion adjacent to the conduction system. It also suggests the significance of electrocardiogram monitoring for over 5 years post - operation in such high - risk patients.

## Introduction

Ventricular septal defect (VSD) is the most common congenital heart disease (1). Transcatheter occlusion of perimembranous ventricular septal defect (Pm-VSD) is an alternative treatment option with limitations in clinical practice (2). In the occlusion of Pm-VSD, recent studies have suggested that the incidence of early CAVB is approximately 0.3%–2.1%, and the incidence of delayed CAVB is 0.3%–0.46% (3–5). Although the incidence of delayed atrioventricular block (CAVB) is not high, its prognosis is poor. Once it occurs, most patients need to be treated with a pacemaker. This article reports a case of persistent third-degree atrioventricular block 7 years after trans-thoracic small-incision eccentric umbrella occlusion, aiming to emphasize the importance of extending the follow-up period for such patients and timely intervention when necessary.

## Case report

**Figure 1:**
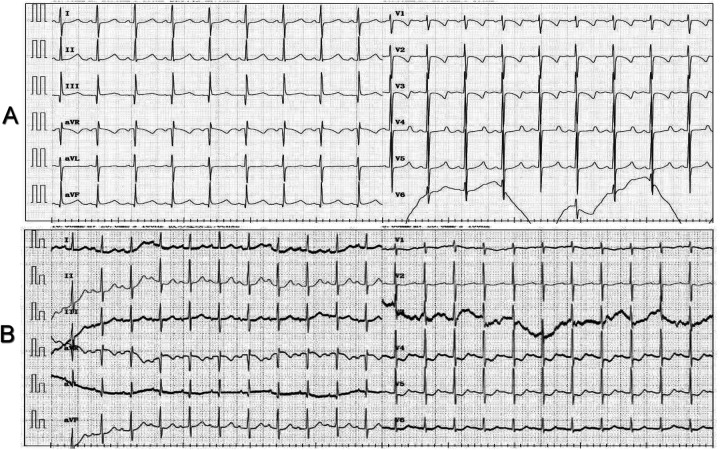
Electrocardiograms before and after surgery. **(A)** Preoperative normal sinus rhythm. **(B)** Early postoperative first-degree AV block.

**Figure 2:**
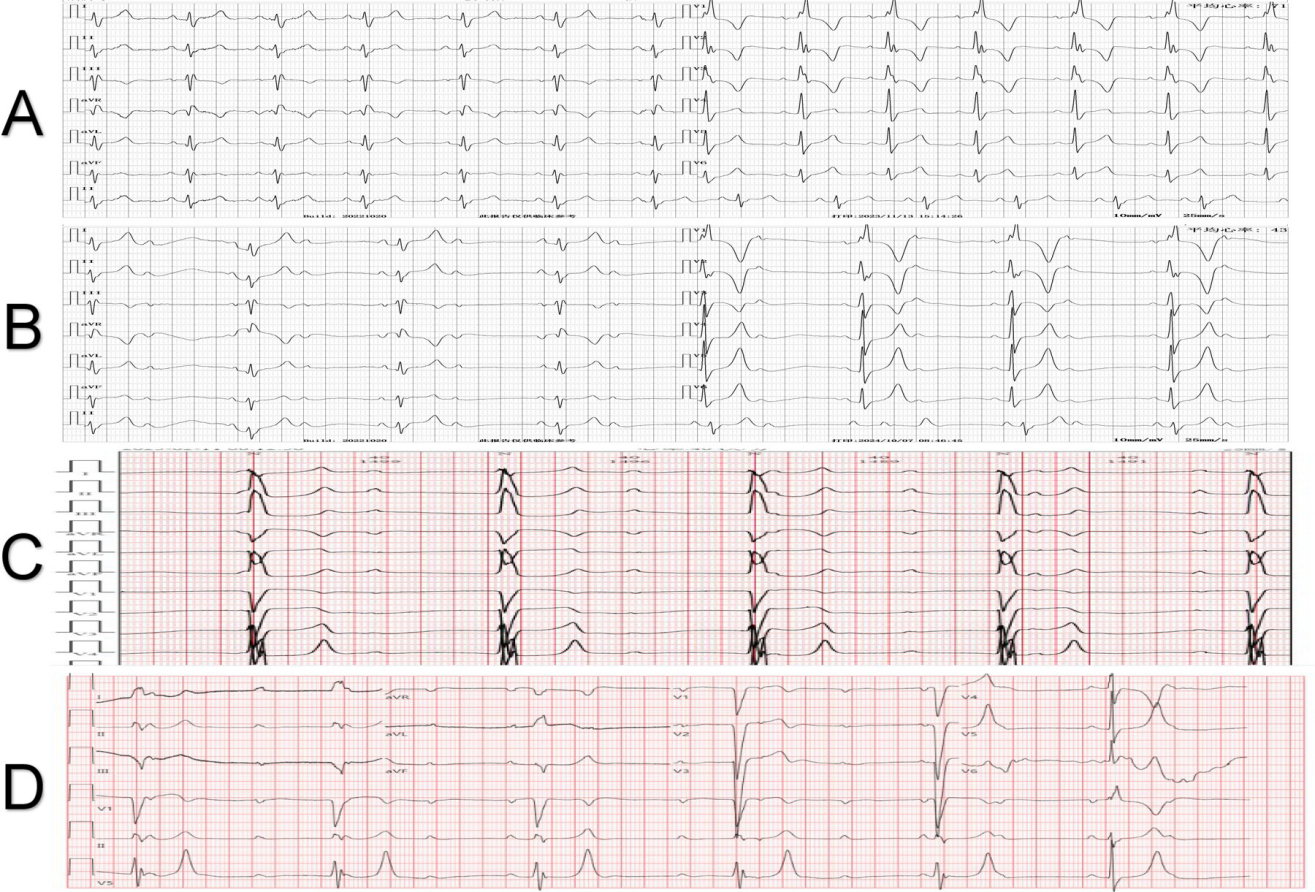
Electrocardiogram manifestations of the patient at different time points after surgery. **(A)** Complete right bundle branch block was shown during the 6-year postoperative follow-up. **(B)** Sinus rhythm with 2:1 atrioventricular block occurred during the 7-year postoperative follow-up. **(C)** Twenty days after the 7-year postoperative follow-up, the electrocardiogram progressed to third-degree atrioventricular block. **(D)** Three months after the removal of the occluder, third-degree atrioventricular block was still present.

**Figure 3:**
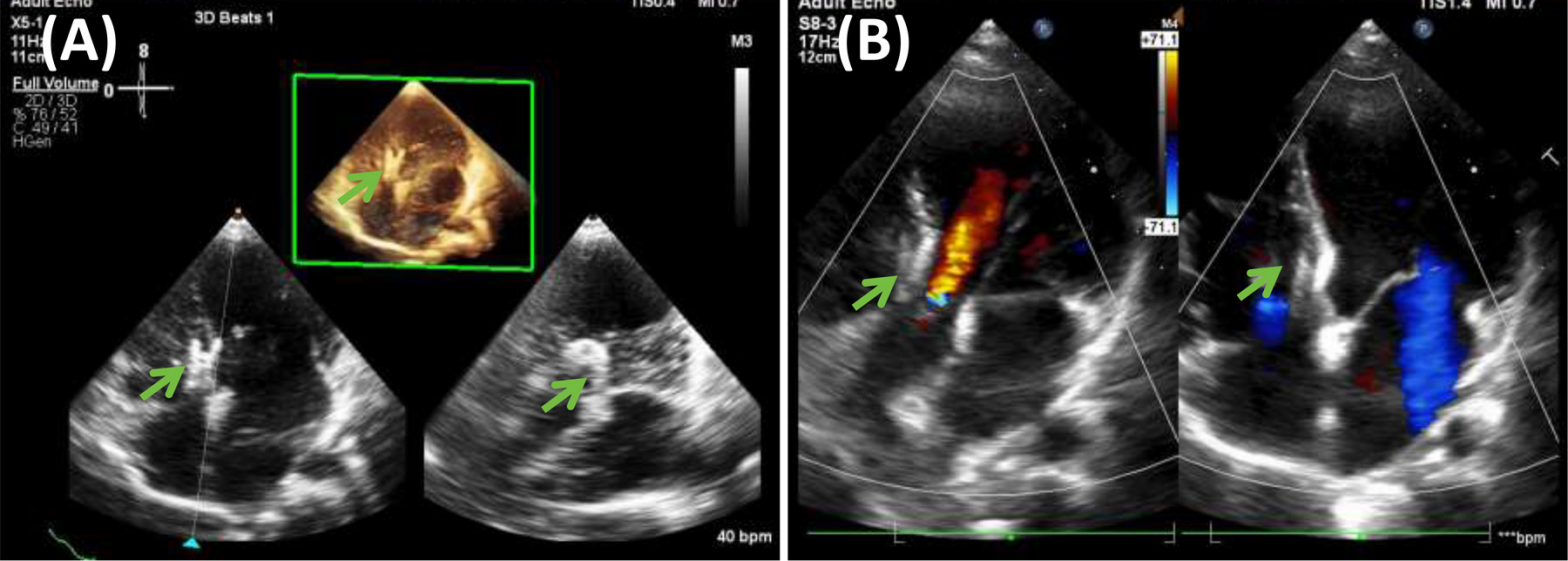
Echocardiogram findings 7 years after surgery. **(A)** Shows the three-dimensional image of the left atrium and ventricle, and **(B)** shows the apical four-chamber and five-chamber view. The area pointed by the green arrow is the eccentric occluder.

The patient was an 8-year-old female who presented with asymptomatic bradycardia detected during a physical examination. Her medical history showed that she had a congenital perimembranous ventricular septal defect (Pm-VSD), and the preoperative electrocardiogram was normal (Figure 1A). In July 2017 (at 4 months of age), preoperative evaluation showed that the defect diameter was approximately 10 mm, the distance from the tricuspid valve margin was ≥2 mm, the distance from the aortic valve was <2 mm, and there was no aortic valve regurgitation. Prior to the surgery, a comprehensive assessment was conducted. Genetic testing for genes such as NKX2.5 was performed, and the results showed no mutations. Also, a meticulous examination for syndromic features associated with TBX5 - related disorders was carried out, but no such features were identified. The family expressed concerns over the potential risks and trauma of traditional open - heart surgery, including large incisions, complications during the operation, and long - term impacts on the child's growth. Given the defect's proximity to the aortic valve, the unique structure of the eccentric umbrella provided a better anatomical fit. Additionally, the trans - thoracic minimally - invasive occlusion with this device promised faster postoperative recovery (2), which was vital for the growth and development of the 4-month-old infant. After in - depth discussions with the medical team, the family requested the trans - thoracic small - incision Pm-VSD occlusion using eccentric umbrella. In addition, relevant studies at that time showed that the clinical application of the eccentric umbrella had good results (6–8). An eccentric umbrella with a diameter of 12 mm was finally used to perform trans-thoracic small-incision Pm-VSD occlusion. After the operation, the patient underwent regular reexaminations. The early electrocardiogram showed a PR interval of 153 ms (Figure 1B), exceeding the normal upper limit (137 ms) for 3–7 month-old females (9, 10), indicating first-degree atrioventricular block, and the ultrasound showed no residual shunt. In November 2023 (the 6th year after the operation), a hospital examination showed a complete right bundle branch block (RBBB) on the electrocardiogram (Figure 2A), and no special treatment was given. In October 2024 (the 7th year after the operation), a slow heart rate of 47 beats per minute was detected during a physical examination, and the electrocardiogram indicated the occurrence of sinus rhythm with 2:1 atrioventricular block (AVB) (Figure 2B). Twenty days later, a reexamination showed progression to third-degree AVB (Figure 2C), and transthoracic echocardiography (TTE) showed significant enlargement of the left atrium and ventricle [left atrial diameter 32 mm (*Z* value =  + 3.2), left ventricular end-diastolic diameter 50 mm (*Z* value =  + 3.5)], normal systolic function (ejection fraction 64%), mild valvular regurgitation, and a stable occluder position with no residual shunt (Figure 3). Subsequently, the occluder was removed and the VSD was repaired, upon removal, it was found that the occluder had been completely endothelialized and was firmly fixed in the body. Immediately after the operation, methylprednisolone 1 mg/(kg·day) was given to reduce myocardial edema, and intravenous infusion of vitamin C (200 mg/day) and sodium phosphocreatine (2 g/day) was used to nourish the myocardium. The drug treatment lasted for 4 days. In February 2025 (more than 3 months of follow-up), the conduction block had not reversed (Figure 2D). The patient refused permanent pacemaker implantation and is currently under continuous follow-up.

## Discussion

**Figure 4:**
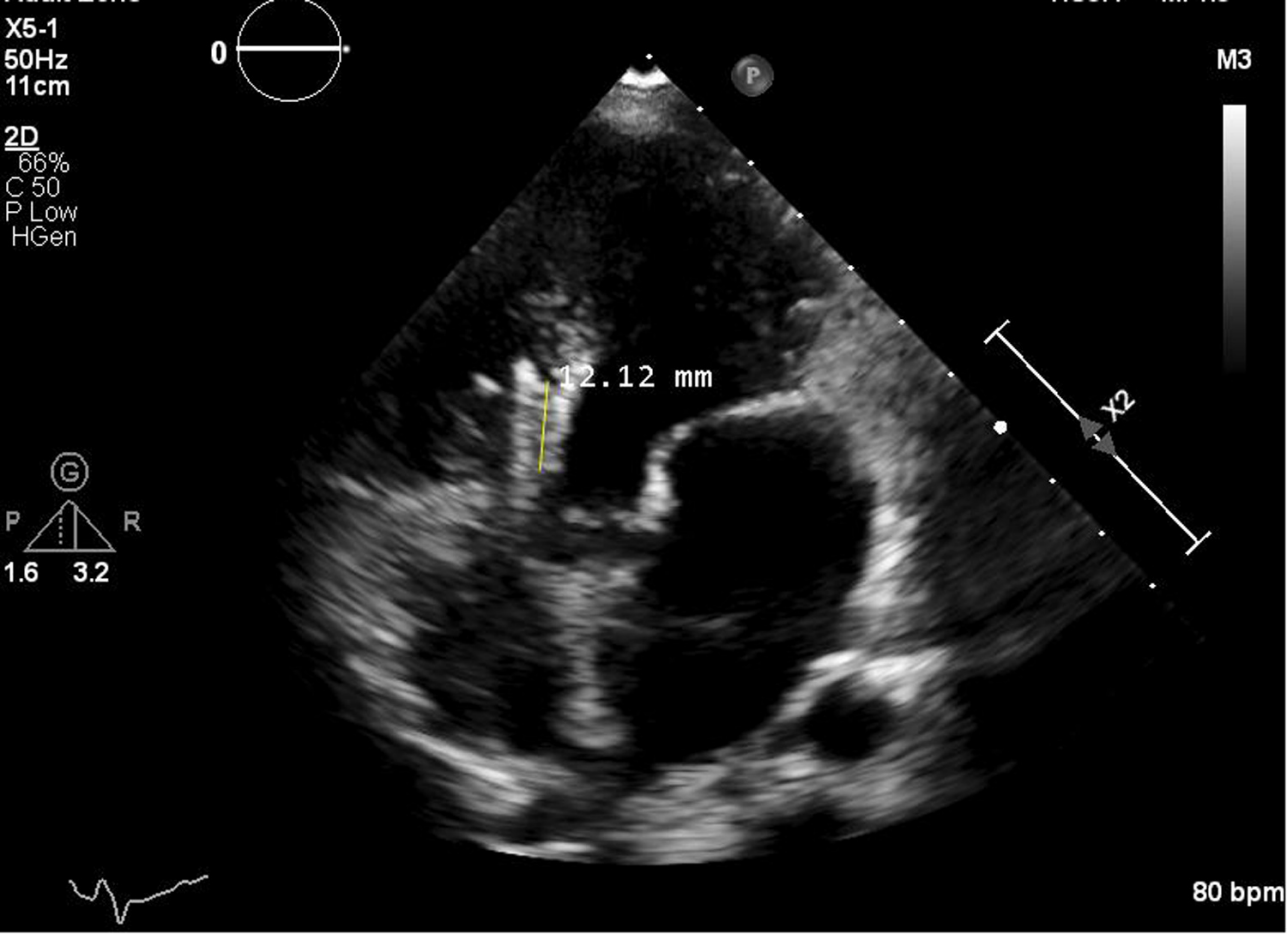
The waist size of the eccentric occluder is shown.

This case presents a rare delayed complication after trans-thoracic occlusion of VSD in an infant. The patient was only 4 months old at the time of occlusion, and the VSD was relatively large, with a diameter of 10 mm, was a significant factor. The selected occluder was also relatively large, with a waist diameter of approximately 12 mm (Figure 4). This is consistent with the correlation between VSD diameter, occluder size, and delayed CAVB reported in previous studies (11). A larger VSD not only indicates more severe structural and hemodynamic issues but also necessitates a larger occluder for closure. Research has shown that VSD diameter is closely linked to the risk of atrioventricular block after occlusion (10). In this case, the 10 - mm defect likely led to the use of a 12 - mm occluder, increasing the risk of mechanical compression on the conduction system. Moreover, the aortic - side flange of the eccentric umbrella's proximity to the conduction bundle further raises this risk. For Pm - VSD treatment, surgical closure is a reliable gold - standard. But for this 4 - month - old with a large, aortic - valve - adjacent Pm - VSD, open - heart surgery had risks like repeated lung infections and extracorporeal - circulation challenges. Considering the family's trauma concerns, trans - thoracic small - incision interventional occlusion with an eccentric umbrella was chosen.

The pathological mechanism is likely associated with early mechanical compression [early postoperative PR interval prolonged to 153 ms, exceeding the normal upper limit of 137 ms (10), indicating compression] and progressive fibrosis. The aortic flange of the eccentric umbrella lies in close anatomical proximity to the His bundle, which further exacerbates this risk. As Jin et al. reported, larger occluders relative to the defect size are associated with an increased risk of post - implant complications, potentially related to mechanical compression on the conduction system (13). Some studies have also shown that delayed CAVB may be related to device-related inflammatory reactions or endothelial hyperplasia, which can lead to the encapsulation of conduction tissues (14, 15). The aortic-side flange of the eccentric umbrella is closer to the conduction bundle, which may increase this risk. It is worth noting that the patient developed complete right bundle branch block (CRBBB) more than 6 years after the operation, and gradually progressed to sinus rhythm with 2:1 atrioventricular block (AVB) and finally to third-degree AVB 7 years after the operation. This disease course evolution may be related to the chronic mechanical compression of the conduction system by the eccentric umbrella and the secondary fibrosis process. This suggests that regular electrocardiogram monitoring after occlusion is crucial, even if the patient is asymptomatic, especially in the first year and even more than 5 years after the operation. According to the European Society of Cardiology Guidelines (2021) (12), if complete left bundle branch block (CLBBB) occurs after occlusion, the occluder should be removed as soon as possible. When CRBBB was detected 6 years after the operation in this case, no special measures were taken, possibly due to insufficient treatment experience. This case further suggests that for patients with the occluder adjacent to the His bundle (especially those who received eccentric umbrella occlusion in infancy), when early conduction bundle abnormalities are detected, timely intervention should be considered, such as removing the occluder and prophylactic pacing, to avoid irreversible damage to the conduction bundle. However, this view still needs to be verified by a large number of clinical studies. In congenital heart disease treatment, the degradable occluder is a new development. It may not suit all patients now but can reduce long - term issues of traditional occluders. Its advantages need more research, and in our paper, we'll focus on its future research potential rather than promoting it as a current alternative. Therefore, in future clinical practice, surgical indications should be more strictly controlled, and appropriate occluders should be selected as much as possible to implement precise treatment. The MemoSorb VSD occluder, launched by Shanghai Shape Memory Alloy Materials Co., Ltd. in 2020, is a fully degradable occluder that can gradually degrade and be absorbed after completing the mission of VSD repair. This feature makes the internal structure of the heart closer to the natural physiological state after the occluder degrades, effectively avoiding a series of complications that may be caused by the long-term retention of nickel-titanium alloy occluders in the body (16). For patients with a defect opening close to the aortic valve (where traditional occluders may compress the conduction bundle after closure and the treatment risk is relatively high), the fully degradable occluder is expected to be a safer treatment option. However, at present, this advantage still needs to be verified by more large-sample and multi-center studies.

## Conclusion

This case exhibits an unusually long interval between trans - thoracic Pm - VSD occlusion and the occurrence of CAVB among reported cases in children under 1 year old, which enriches our understanding of the long - term outcomes of such procedures. For high - risk patients with occluders near the conduction system, it implies the importance of extended electrocardiogram monitoring, and the need for more research. It not only emphasizes the necessity of long-term electrocardiogram monitoring but also reveals the potential risks related to the design of the eccentric umbrella. Further research is needed to clarify the predictive factors of delayed conduction abnormalities and optimize the long-term management strategy (especially whether to intervene in a timely manner when subtle conduction bundle abnormalities are detected). This case serves as a wake-up call for clinicians to conduct long-term follow-up of such patients and provides an important reference for continuously exploring the field of congenital heart disease treatment to improve patient prognosis and safety.

## Data Availability

The original contributions presented in the study are included in the article/Supplementary Material, further inquiries can be directed to the corresponding author.
